# The life in a gradient: calcium, the lncRNA SPRR2C and mir542/mir196a meet in the epidermis to regulate the aging process

**DOI:** 10.18632/aging.203385

**Published:** 2021-08-02

**Authors:** Sven Breunig, Veronika Wallner, Katharina Kobler, Herbert Wimmer, Peter Steinbacher, Maria Karolin Streubel, Johannes Bischof, Jutta Duschl, Claudia Neuhofer, Wolfgang Gruber, Fritz Aberger, Michael Breitenbach, Elisabeth Russe, Gottfried Wechselberger, Albert Duranton, Klaus Richter, Mark Rinnerthaler

**Affiliations:** 1 Procomcure Biotech, Breitwies, Thalgau, Austria; 2 Department of Biosciences, Paris-Lodron University Salzburg, Salzburg, Austria; 3 EB House Austria, Research Program for Molecular Therapy of Genodermatoses, Department of Dermatology and Allergology, University Hospital of the Paracelsus Medical University Salzburg, Salzburg, Austria; 4 Department of Biosciences, Cancer Cluster Salzburg, Paris-Lodron University Salzburg, Salzburg, Austria; 5 Department of Plastic and Reconstructive Surgery, Hospital of the Barmherzige Brüder, Paracelsus Medical University Salzburg, Salzburg, Austria; 6 L'Oréal Research and Innovation, Aulnay-sous-Bois, France

**Keywords:** miRNA, lncRNA, pseudogene, skin aging, epidermal barrier

## Abstract

The turnover of the epidermis beginning with the progenitor cells in the basal layer to the fully differentiated corneocytes is tightly regulated by calcium. Calcium more than anything else promotes the differentiation of keratinocytes which implies the need for a calcium gradient with low concentrations in the stratum basale and high concentrations in the stratum granulosum. One of the hallmarks of skin aging is a collapse of this gradient that has a direct impact on the epidermal fitness. The rise of calcium in the stratum basale reduces cell proliferation, whereas the drop of calcium in the stratum granulosum leads to a changed composition of the cornified envelope. We showed that keratinocytes respond to the calcium induced block of cell division by a large increase of the expression of several miRNAs (hsa-mir542-5p, hsa-mir125a, hsa-mir135a-5p, hsa-mir196a-5p, hsa-mir491-5p and hsa-mir552-5p). The pitfall of this rescue mechanism is a dramatic change in gene expression which causes a further impairment of the epidermal barrier. This effect is attenuated by a pseudogene (SPRR2C) that gives rise to a lncRNA. SPRR2C specifically resides in the stratum granulosum/corneum thus acting as a sponge for miRNAs.

## INTRODUCTION

The aging process is to a large part still enigmatic. This is attributed to the fact that aging is multifactorial and is dependent on such processes as senescence, loss of proteostasis, changes on the epigenetic level, altered mitochondrial functions and many more (reviewed in [1]). The aging process in the skin is even more complex, because this tissue consists essentially of two layers (epidermis and dermis) that is predominantly populated by two different cell types: keratinocytes and fibroblasts, respectively. If a hallmark of an aged dermis has to be named, it is the changed architectural organization of the extracellular matrix, finally resulting in folds and wrinkles. The dermis is also a wonderful example for the force of extrinsic aging. Increased UV-exposure (′photoaging′) results in increased ROS levels that stimulates the activation of matrix metalloproteases thus leading to an increased turnover of the extracellular matrix [2].

One of the hallmarks of an aged epidermis, however, is the breakdown of the epidermal calcium gradient [3, 4]. Calcium is a key driver for keratinocyte differentiation and thus for the formation of the epidermal barrier. On the one hand, this barrier protects us against environmental influences such as invading microorganisms, xenobiotics, pollutants and mechanical stress; on the other hand, it reduces the transepidermal water loss. The protective effect of the epidermis is built on three pillars: (1) a heavily cross-linked protein network (cornified envelope; the “bricks” of the epidermis): (2) lipids as a multilamellar barrier between the cells (mainly ceramides; the “mortar” of the epidermis); and (3) the acidic skin pH [5]. The small ion calcium promotes the expression of most cornified envelope specific proteins including involucrin, fillagrin, loricrin and the small proline rich repeat (SPRR) family [6–8]. All these proteins are crosslinked by the formation of Nɛ-(γ glutamyl) lysine (isopeptide) bonds in a Ca^2+^-dependent manner [9, 10]. In addition, the secretion of the lipid filled “lamellar bodies” and thus the formation of the “mortar” between the keratinocytes is driven by calcium [11].

Despite its undeniable essential functions in the epidermis, high concentrations of this ion can be detrimental. Ca^2+^ leads to fully differentiated keratinocytes (corneocytes) in the *stratum corneum* and cell death through a highly regulated cell death pathway. Accordingly, calcium would be a high risk factor for the progenitor cells that reside in the *stratum basale*. To cope with such different demands for calcium a gradient is established. The Ca^2+^ concentration is lowest in the *stratum basale* (cells attached to the basal lamina; in immediate proximity to the dermis) and constantly rises from the *startum spinosum* to the *stratum granulosum*, where a peak is reached. However in the *stratum corneum,* the outmost layer of the epidermis, a clear drop of calcium can be observed [12]. During aging the overall amount of calcium in the epidermis is not decreasing, but the epidermal calcium gradient is collapsing. This has two consequences for the epidermis: A lowered calcium concentration in the *stratum granulosum* and an increased Ca ^2+^-level in the *stratum basale*. This redistribution leads to a multitude of side effects: The cornified envelope gets restructured, the epidermal barrier gets rigid and the skin pH rises [3, 8, 13]. It is quite possible that another prominent phenotype of skin aging is attributed to the redistribution of calcium. The skin turnover (the shedding of dead corneocytes and its replacement) is markedly increased from ~30 days to ~60 days [14]. This phenomenon is mainly attributed to the so called Hayflick limit, but it is also likely that the increased leakage of calcium into the *stratum basale* is involved in the reduced renewal of keratinocytes.

In this paper we are not considering factors that contribute to the breakdown of the epidermal calcium gradient, but we demonstrate how the epidermis adapts to the altered calcium levels in each *stratum* by the expression of lncRNAs and miRNAs.

## RESULTS

**Figure 1:**
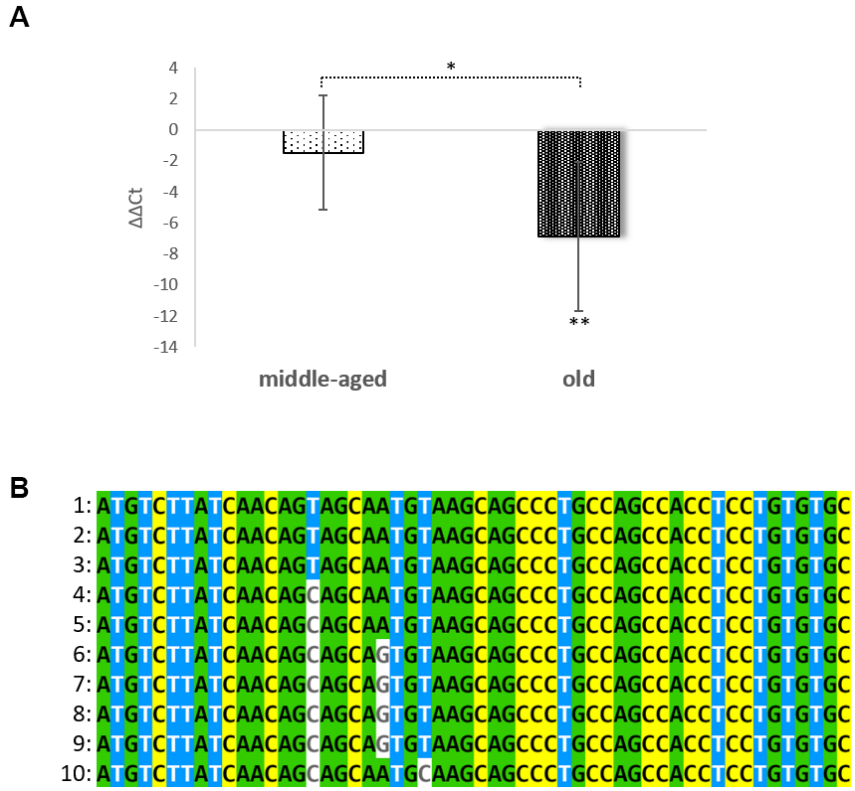
**Age dependent expression of SPRR2C and conservation in evolution.** In (**A**) the ΔΔCt values of SPPR2C in foreskin samples of middle aged (age range: 24-39 years) and elderly people (age range: 60 to 76 years) are shown after normalization to young foreskin samples (age range: 2.5-8 years), (N=5 in each group;). One-way ANOVA analysis indicates a significant difference between groups (p= 0.0153285). In (**B**) a multiple sequence alignment (first 54 nucleotides of the ORF) of SPRR2C in hominids, apes and mammals was calculated using MVIEW (https://www.ebi.ac.uk/Tools/msa/mview/). In most hominids the 6^th^ codon (CAG) encodes a glutamine but is changed into a STOP codon (TAG) in *Homo sapiens*, *Pan troglodytes* and *Pan paniscus*. 1: *Homo sapiens*; 2: *Pan troglodytes*; 3: *Pan paniscus*; 4: *Gorilla gorilla gorilla*; 5: *Pongo abelii*; 6: *Sapajus paella*; 7: *Papio Anubis*; 8: *Cebus capucinus*; 9: *Chlorocebus sabaeus*; and 10: *Condylura cristata*.

Previously, we were able to demonstrate that the cornified envelope is rebuilt during the aging process as a direct response to the collapsing calcium gradient. These changes include such hallmarks as down-regulation of loricrin (the most prevalent protein in the CE) and an upregulation of most small proline rich repeat proteins [3, 15, 16]. It has to be stated that the so called “CE precursor family“ has its origin in a single progenitor gene and presumably arose due to a series of gene duplications [17]. Therefore, all members of this family (loricrin, involucrin, SPRR1A-1B, SPRR2A-2G, SPRR3, SPRR4, LCE1A-1F, LCE2A-2D, LCE3A-3E, LCE4A-6A and LEP7) are located in the epidermal differentiation complex on the chromosomal region 1q21 [18, 19]. This family also harbors a long non-coding RNA (lncRNA): SPRR2C. During evolution, a C at position 102 was mutated into a T. In this way the 6^th^ codon (glutamine) was changed into a STOP (TAG). Careful re-evaluation of a series of microarray data using Gene Chip HGU133 Plus 2.0 Arrays (Affymetrix) [15] showed a differential expression of this pseudogene. In this study five non-inflamed foreskin tissues from middle aged individuals (age: 18 years – 28 years) were compared with foreskin samples obtained from elderly people (58 years to 74 years). The analysis revealed a 13.96-fold up-regulation of SPRR2C (p-value=0.016). When the same aged foreskin samples were compared with three young non-inflamed foreskin samples (3-5 years) (Rinnerthaler et al., unpublished data) a 8.99-fold up-regulation with increasing age was observed (p-value: 0.071). A RT-PCR analysis comparing young, middle aged and old foreskin samples confirmed the strong increase in SPRR2C expression with age (Figure 1A). On average the SPRR2C levels in old people were 116-fold and 41-fold increased compared to young and middle-aged skin samples, respectively. The rise of this lncRNA in some elderly is dramatic (more than 1050-fold). The presence of this C to T transition resulting in a premature STOP codon is not specific for humans but occurred late in hominid evolution (Figure 1B). Because humans as well as chimpanzees and bonobos have this pseudogene, it is very probable that there is a certain evolutionary advantage in keeping the pseudogene.

**Figure 2:**
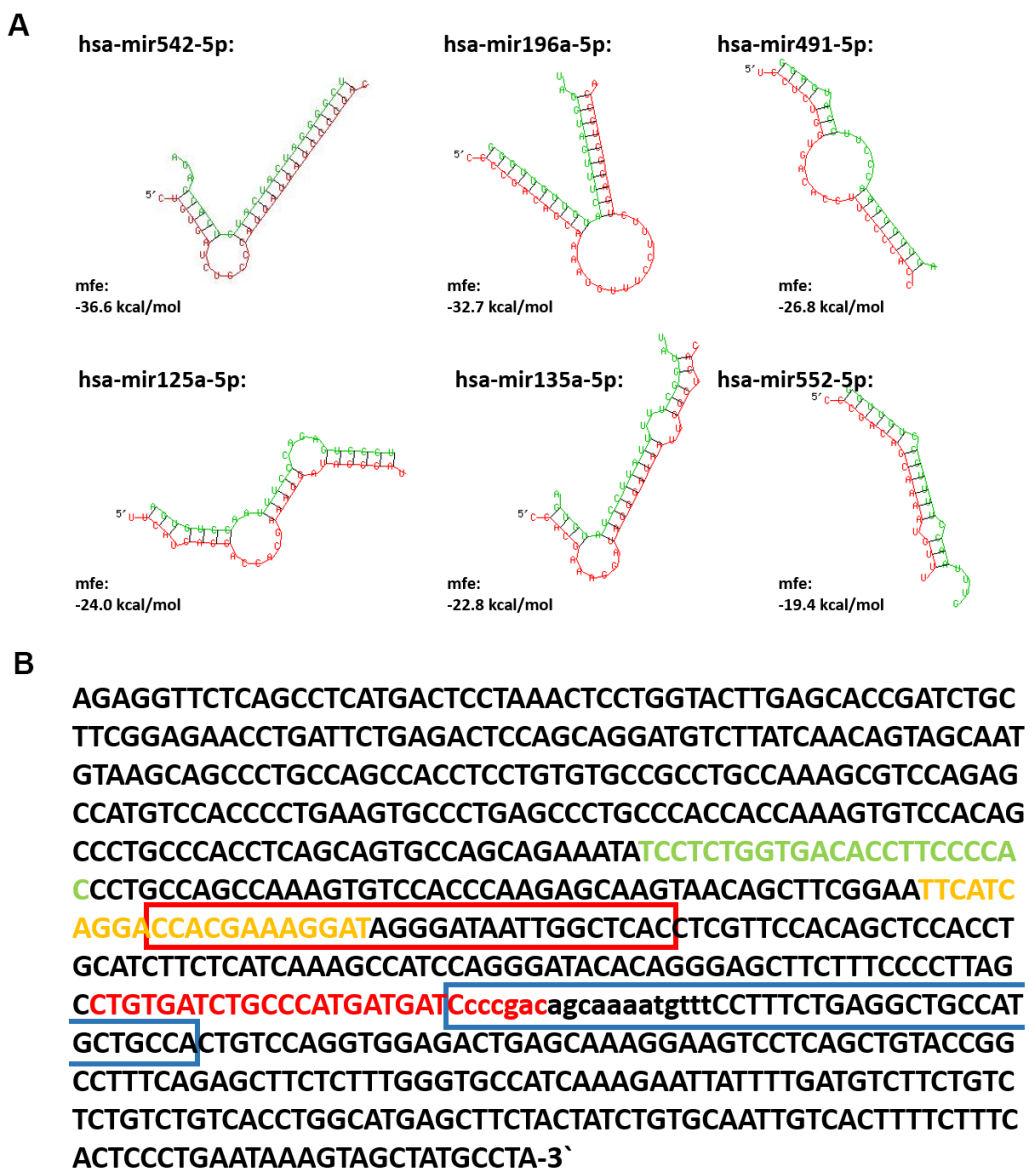
**The interaction between lncRNA SPRR2C and several miRNAs.** In (**A**) the SPRR2C-miRNA hybrids are shown. The SPRR2C region is presented in red, the appropriate miRNA in green. Both RNA-miRNA hybrids and mfe were calculated using RNAhybrid (https://bibiserv.cebitec.uni-bielefeld.de/rnahybrid). In (**B**) the SPRR2C mRNA is presented and the binding sites for hsa-mir542-5p, hsa-mir125a, hsa-mir135a-5p, hsa-mir196a-5p, hsa-mir491-5p and hsa-mir552-5p are marked. hsa-mir542-5p: red letters; hsa-mir125a-5p: yellow letters; hsa-mir135a-5p: red bracket; hsa-mir196a-5p: blue bracket; hsa-mir491-5p: green letters; hsa-mir552-5p: lower case letters.

In several publications a role for lncRNAs as ‘miRNA sponges’ was suggested [20–22]. Accordingly, SPRR2C was already identified as a miRNA-196a-5p target [23]. By using several online available tools, the most promising miRNAs were predicted that are potentially binding to the lncRNA SPRR2C. According to this analysis, the best candidates are hsa-mir542-5p, hsa-mir125a, hsa-mir135a-5p, hsa-mir196a-5p, hsa-mir491-5p and hsa-mir552-5p. The minimal free energy (mfe) for the hybridization between the miRNAs and their target SPRR2C was recalculated by using RNAhybrid [24]. In all cases the seed regions of the predicted miRNAs (nucleotides 2/3-7/8 from the 5′-end) perfectly match SPRR2C (Figure 2A). The potential binding sites of these 6 miRNAs in the SPRR2C sequence are shown in Figure 2B. The two best hits according to our analysis were hsa-mir542-5p and hsa-mir196a-5p with a mfe of -36.6 kcal/mol and -32.7 kcal/mol, respectively. Attributed to the ancestral origin most members of the “CE precursor family“ are targets of the same miRNAs (Supplementary Figure 1).

**Figure 3:**
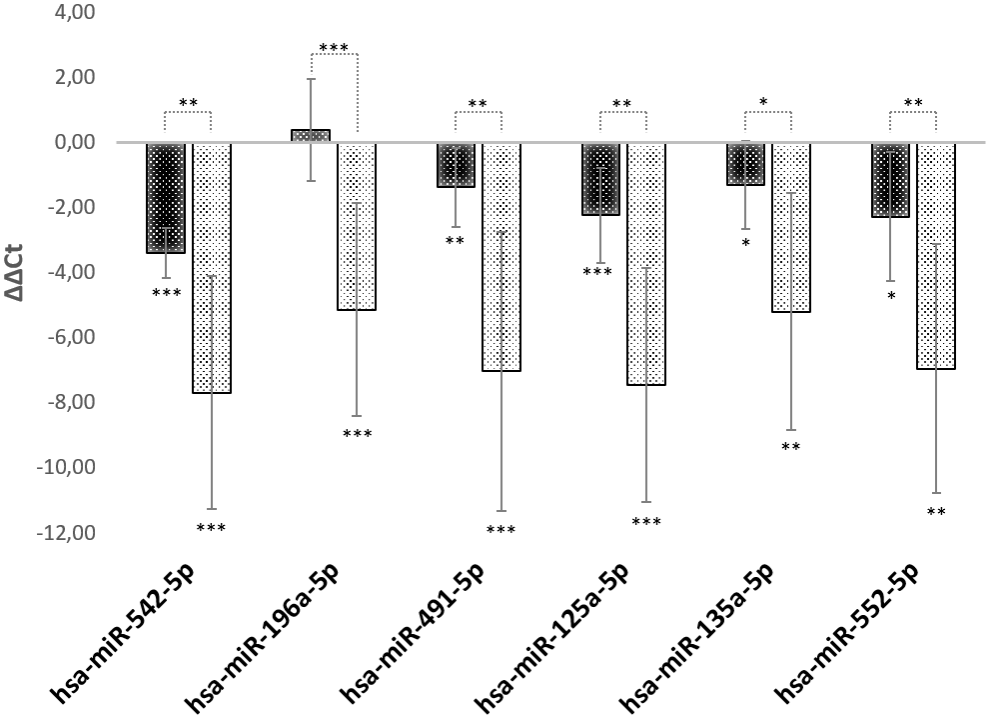
**miRNA expression levels during aging.** RT-PCR analysis of miRNAs specifically targeting SPRR2C. The ΔΔCt values for hsa-mir542-5p, hsa-mir125a-5p, hsa-mir135a-5p, hsa-mir196a-5p, hsa-mir491-5p and hsa-mir552-5p are presented. All values are normalized to young foreskin (age range: 2.5-8 years). Black columns: middle aged foreskin samples (age range: 24-39 years); grey columns: old foreskin samples (age range: 60 to 76 years). (N=5 in each group; *: p<0.1; **: p< 0.05; ***: p<0.01).

Using a RT-PCR approach, we analyzed the regulation of hsa-mir542-5p, hsa-mir125a-5p, hsa-mir135a-5p, hsa-mir196a-5p, hsa-mir491-5p and hsa-mir552-5p in young, middle-aged and old foreskin samples (N=5 of each age). The exact same foreskin samples were used as in Figure 1A and data are presented in Figure 3. It has to be stated that the levels of all miRNAs that were tested show a dramatic continuous increase with age. The expression levels of mir542-5p rise more than 10-fold on the transition from childhood to adolescence. In old age, a more than 200-fold up-regulation of mir542-5p was observed. The fold-increase for all other miRNAs is as follows: 35-fold (hsa-miR-196a-5p); 132-fold (hsa-miR-491-5p); 176-fold (hsa-miR-125a-5p); 37-fold (hsa-miR-135a-5p) and 124-fold hsa-miR-552-5p. In most of the skin samples a high SPRR2C expression level correlates very well with a high abundance of hsa-mir542-5p (Pearson Correlation Coefficient R=0.7967).

**Figure 4:**
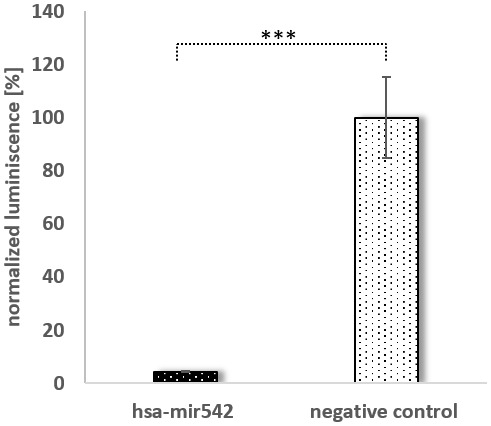
**The binding of mir542-5p to its target SPRR2C.** A dual luciferase assay was performed by sequentially measuring firefly and renilla luciferase. A immortalized keratinocyte cell line was transfected with 350 ng psiCHECK2**™**-SPRR2C (281-486) and either 125 nM MISSION miRNA MIMIC hsa-mir542 or MISSION miRNA MIMIC Negative Control #1. Black bars indicate a co-transfection with mir-542-5p, grey bars a co-transfection with the control miRNA. All data are normalized to the firefly luciferase activity (N=4). An independent t-test analysis indicated a significant difference.

For the following experiments, we focused on two miRNAs: mir196a-5p as a *bona fide* SPRR2C binding miRNA [23]; miR542-5p due to its high expression and low mfe. The SPRR2C binding of mir542-5p and mir196a-5p was validated as follows: Either full length SPRR2C or a specific region of SPRR2C (nucleotides 407-628) were cloned into the luciferase reporter plasmid psiCHECK™-2. The latter region was chosen, because it harbors most miRNA binding sites (Figure 2B). In mammalian cells two RNAs are produced from this construct: (1) a firefly luciferase mRNA used for normalization; (2) a renilla luciferase (downstream)-SPRR2C (upstream) hybrid mRNA. The stability of the hybrid mRNA was tested via RT-PCR. For this assay HaCaT cells were co-transfected with either a negative control miRNA or hsa-mir542-5p or hsa-mir196a in combination with one of the three psiCHECK™-2 vectors (psiCHECK™-2; psiCHECK™-2-SPRR2C; psiCHECK™-2-SPRR2C (281-486 fragment)). The successful transfection with the miRNA was verified by RT-PCR analysis with specific primers after RNA isolation and poly-A-tailing (data not shown). In a first step the RNA levels of the renilla luciferase in relation to the firefly luciferase mRNA was analyzed via RT-PCR. Confirming the results of Maru et al. [23] we saw a decrease of renilla luciferase levels that were expressed from the psiCHECK™-2-SPRR2C (407-628) vector after addition of mir196a-5p (data not shown). However, it has to be stated that in our hands the mir196a effect on luciferase expression was dramatically less (a 10% decrease compared to a 70% decrease as seen by Maru). In case of mir542-5p transfection (40 nM) a highly significant 20% decrease of renilla luciferase abundance expressed from the psiCHECK™-2-SPRR2C (407-628) vector was detected. This reduction was even confirmed at lower mir-542-5p concentrations albeit at lower levels indicating a concentration dependent degradation of SPRR2C (data not shown). By using a commercially available assay measuring dual luciferase activity (renilla luminescence compared to firefly luminescence) we saw a nearly complete abolishment of renilla luciferase activity (~23-fold reduction in activity) after transfection with 125 nM mir-542-5p (Figure 4). In cells co-transfected with the control vector psiCHECK™-2 and mir-542-5p no downregulation of renilla luciferase activity was observed.

**Figure 5:**
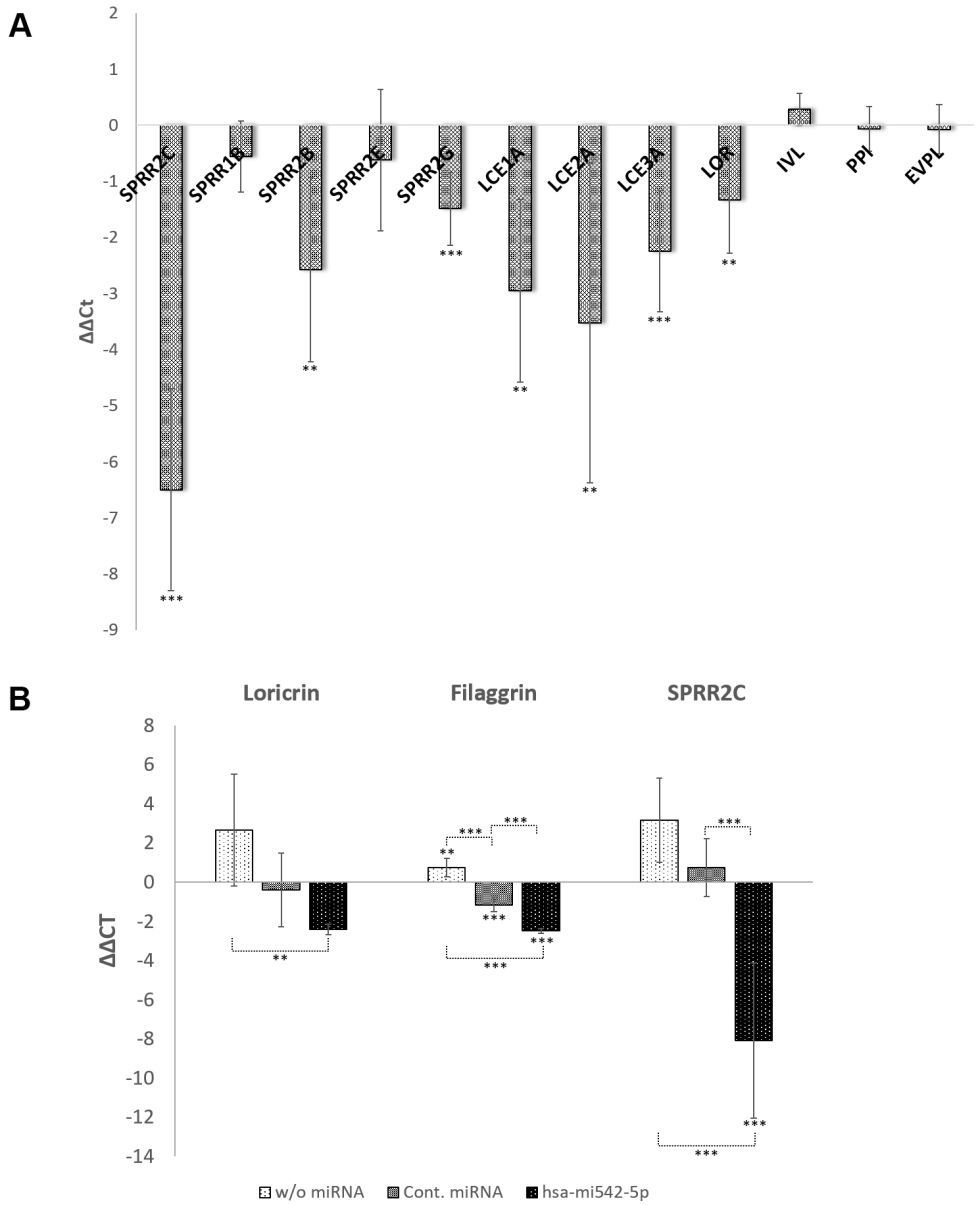
**The role of the lncRNA SPRR2c and hsa-mir542-5p in keratinocyte differentiation.** RT-PCR analysis of selected CE family members are presented as ΔΔCt values in (**A**, **B**). In (**A**) SPRR2C (pLL3.7-SPRR2c) is overexpressed after transduction of HaCaT cells with lentiviral particles. In (**B**) the increase of expression of several CE family members is analyzed after addition of 0.8 mM CaCl_2_ and transfection of HaCaT cells with hsa-mir542a-5p (N=4; *: p<0.1; **: p<0.05; ***: p<0.01).

In a next step the effect of a SPRR2C overexpression on keratinocyte differentiation and cornified envelope formation was tested. SPRR2C was cloned into the retroviral transfer vector pLL3.7 [25] and after lentiviral production in HEK293FT cells, HaCaT cells were transduced with the lncRNA. Using qPCR the overexpression of SPRR2C was confirmed (~90-fold; Figure 5). In addition, the expression level of selected CE specific genes was tested. The overexpression of the pseudogene SPRR2C resulted in an increase of LCE2A (11.5-fold), LCE1A8 (7.7-fold), SPRR2B (6-fold), LCE3A (4.8-fold), SPPR2G (2.8 -fold) and loricrin (2.5-fold). The positive effect of the lncRNA SPRR2C was confirmed by Western blot analysis. Previously, SPRR2A was already established as a marker for keratinocyte differentiation that responds to skin aging and were thus chosen for this study [3]. In fact, SPRR2C overexpression also results in an increase in SPRR2A protein levels (Supplementary Figure 2).

The transfection of HaCaT cells with mir196a-5p had the opposite effect, although on modest levels. In case that a knock-down of SPRR2C was observed, the differentiation status of keratinocytes decreased as deduced from loricrin expression levels (in the mean a 2-fold reduction; the Pearson correlation coefficient R between SPRR2C and loricrin expression is 0.88; N=6). Surprisingly, the transfection with mir542-5p showed no correlation between SPRR2C and loricrin levels (R=-0.30). In each single experiment (N=6) mir542-5p induced a SPRR2C response ranging from a 19.7-fold up-regulation to a 5.5-fold down-regulation.

**Figure 6:**
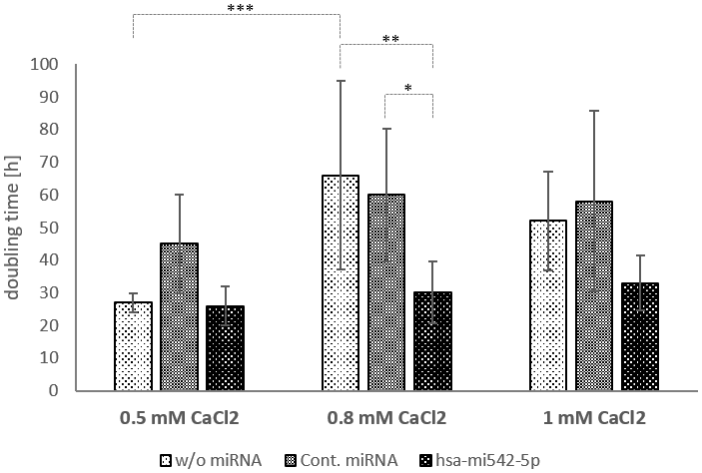
**The doubling time of HaCaT cells in dependence of calcium and mir542-5p.** HaCaT cells were treated with 0.5, 0.8 and 1 mM CaCl2 to induce the formation of the cornified envelope. In parallel cells were transfected with a control miRNA and hsa-mir542-5p. Cell numbers were calculated 24, 48 and 72 hours after start of treatment (N=4-6; *: p<0.1; **: p<0.05; ***: p<0.01).

Therefore, another approach was chosen to test the involvement of mir542-5p in keratinocyte differentiation. Ca^2+^ is a key player in the transition from keratinocytes to corneocytes and thus reduces the cell division rate. In fact, addition of 0.8 mM CaCl_2_ increased the doubling time of HaCaT cells from 27 hours (0.5 mM CaCl_2_) on average (N=4) to 66 hours (N=6; p-value=0.0308). No significant further change in doubling time was observed by rising the calcium concentration to 1mM CaCl_2_ (Figure 6). Transfection of HaCaT cells with mir542-5p completely restored the cell division rate (30 hours at 0.8 mM CaCl_2_; 33 hours at 1 mM CaCl_2_). Based upon the finding that mir542-5p specifically counterbalances the effects of high calcium concentrations, the role of this miRNA was tested at increased Ca^2+^ levels. At 0.8 mM CaCl_2_ (compared to 0.09 mM CaCl2) a more than 6-fold rise of loricrin levels was observed (Figure 5B). This confirms previously published loricrin expression levels [26]. Additionally, we also detected 9-fold increased SPRR2C expression levels upon addition of 0.8 mM CaCl_2_. After transfection with mir542-5p, SPRR2C, loricrin and filaggrin levels decreased: (1) SPRR2C: 272-fold down; (2) loricrin: 5-fold down; and (3) filaggrin: 5.6-fold-down. In accordance to results presented above, the mir542-5p effects on CE formation are less pronounced at lower CaCl_2_ concentrations (data not shown). It also has to be stated that the transfection process itself had some effect on the differentiation status of keratinocytes (Figure 5B).

**Figure 7:**
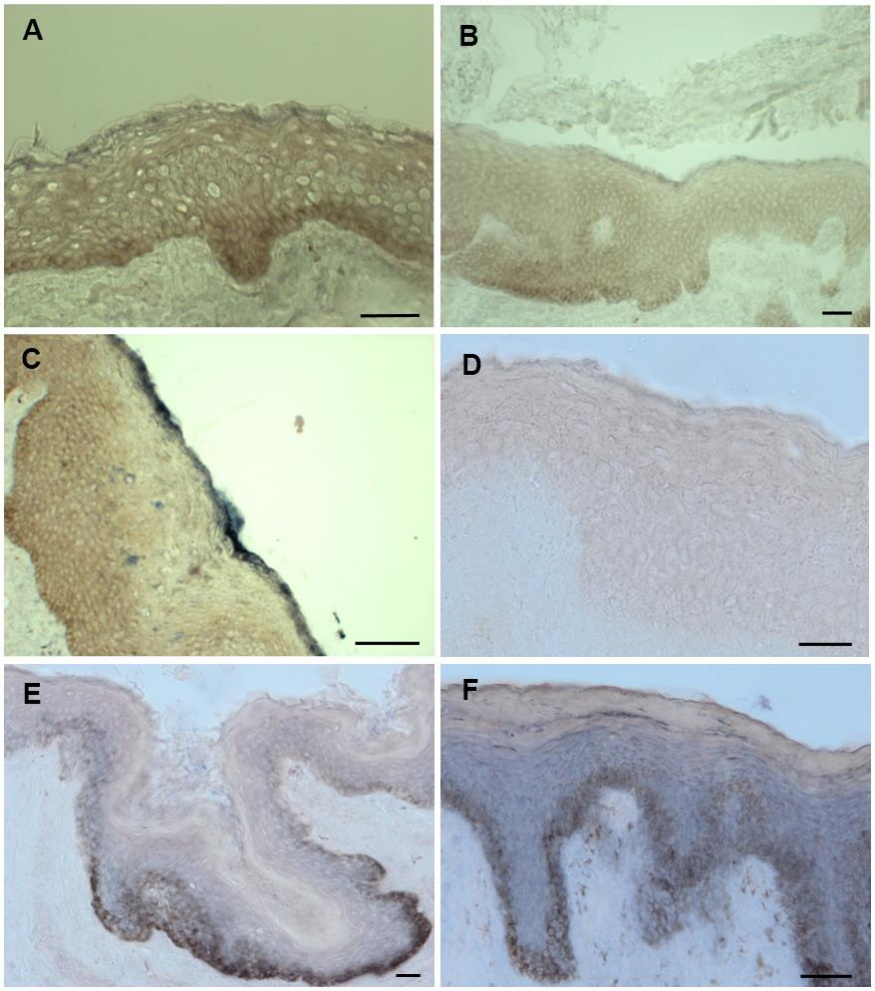
**ISH staining of SPRR2c and mir542-5p.** (**A**–**C**) ISH staining of SPRR2C. (**A**) Foreskin sample from a 6-year-old; (**B**) Foreskin sample of a 30-year-old; (**C**) Foreskin sample of a 61 year old. Increased SPRR2C levels were detected in old skin samples compared to young skin. (**D**–**F**) ISH staining of mir542-5p. (**D**) Foreskin sample of an eight-year old; (**E**) Foreskin sample of a 30-year-old; (**F**) Foreskin sample of a 61-year old. During aging the hsa-mir542-5p levels gradually increase. (**B**, **E**) as well as (**C**, **F**) are specimen from identical donors. The intensity of the black-purple staining correlates with the amount of either SPRR2C or mir-542-5p. *Stratum basale* appears brownish due to the presence of melanocytes and is no specific staining. Scale bar: 50 μm.

In the epidermis ions, RNAs as well as proteins are organized in a gradient. Therefore, it is of utmost importance that the antagonists mir542-5p and SPRR2C meet in some epidermal strata. In a RNA ISH (*in situ* hybridization) approach both SPRR2C and mir542-5p where localized in human skin samples by digoxygenin labelled probes. Most CE proteins are synthesized in and localize to the outermost *stratum spinosum*, *stratum granulosum* and *stratum corneum*. Although SPRR2C is not encoding a protein, the localization of the lncRNA is not unusual and can be found in the *stratum granulosum* and *corneum* (Figure 7B, 7C). In addition, the RNA *in situ* hybridization experiments confirm the RT-PCR results (Figure 1A). SPRR2C is hardly expressed in young foreskin samples and shows increased levels from middle-aged to old individuals. In Figure 7A–7C representative images are shown. The expression and localization of SPRR2C is not specific for the body region and was observed in foreskin as well as eyelid samples. The exact same phenotypes were observed for eyelid samples obtained from middle-aged and old women after blepharoplasty (data not shown). RNA ISH experiments also show an increase in mir542-5p levels in aged skin samples (Figure 7D, 7E) and thus confirm a previous RT-PCR analysis (Figure 3). The expression of mir542-5p is equally distributed across all epidermal strata with the exception of *stratum corneum*. In oversaturated specimen, mir542-5p was also observed in *stratum corneum* (data not shown). No traces of this miRNA can be localized in the dermis and it seems to be epidermal specific. In young skin samples mir542-5p is practically undetectable. The RNA ISH experiments demonstrate that both mir542-5p and SPRR2C meet in the stratum *granulosum* and *corneum*.

## DISCUSSION

**Figure 8:**
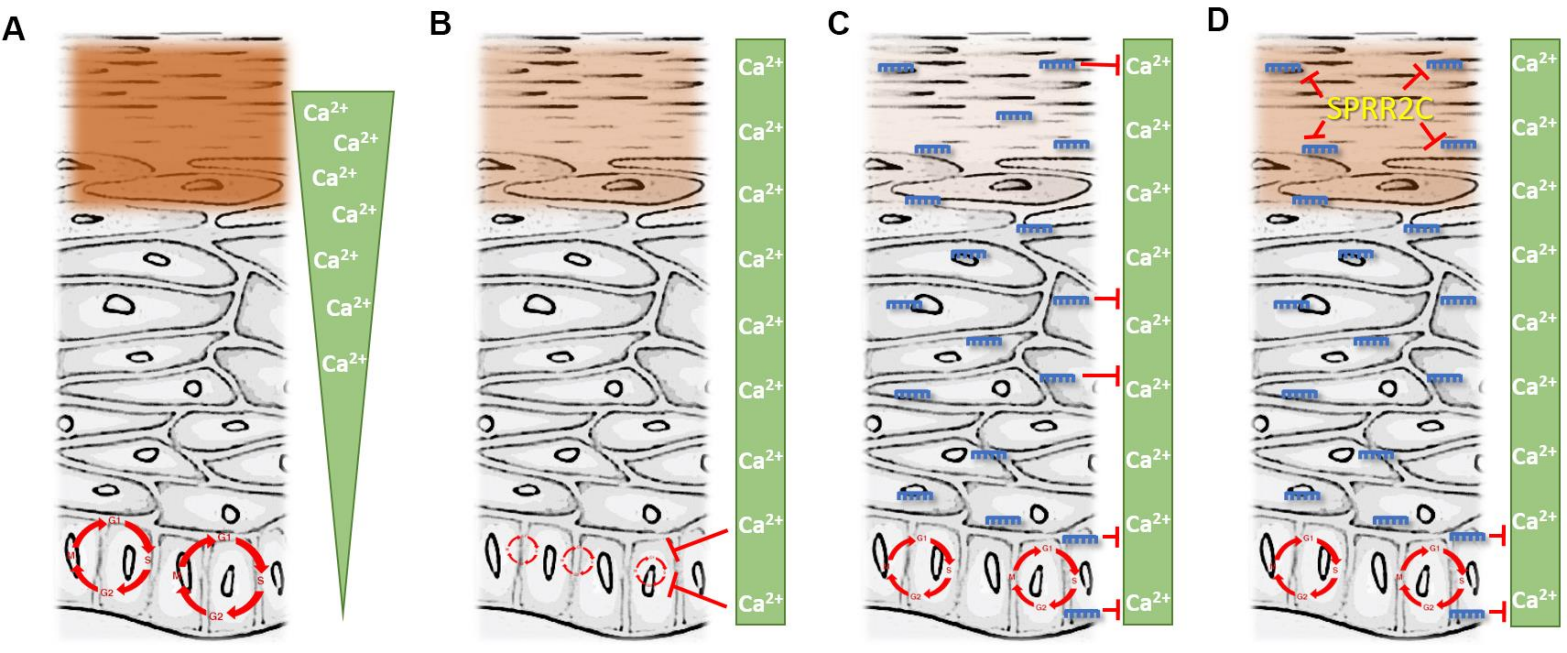
**The interplay between the lncRNA SPRR2c and several miRNAs.** (**A**) represents the situation in young and healthy skin. (**D**) is representative for aged skin. (**B**, **C**) are hypothetical situations with either the absence of miRNA (**B**) or SPRR2C (**C**). Green triangle: epidermal calcium gradient; green box: evenly distributed calcium after the collapse of the epidermal calcium gradient (e.g. during skin aging). Red circle: Cell cycle. Size indicates the cell proliferation rate (the bigger the faster). Brown box: cornified envelope (the intensity is correlated with the amounts of proteins in the CE). miRNAs: blue; lncRNA: yellow.

The central findings of this paper are summarized in Figure 8. In healthy epidermis a calcium gradient is established. This is of absolute necessity because Ca^2+^ is essential for differentiation of keratinocytes and therefore detrimental to progenitor cells. The highest amounts of Ca^2+^ can be found in the *stratum granulosum*. In this epidermal layer as well as in the outermost *stratum spinosum* most members of the CE precursor family as well as ω-OH ceramides are formed in a calcium dependent manner. All these proteins get cross-linked and form the cornified envelope, a multi-protein mesh below the plasma membrane. Additionally, the phospholipids in the plasma membrane get replaced by ceramides and thus the keratinocytes transform into corneocytes that are finally shed. All the processes listed above are in strict dependence on high amounts of calcium. These facts also explain that calcium is a serious threat to the progenitor cells in the *stratum basale*, the basic layer of the epidermis, due to the induction of the differentiation process. Therefore, the calcium concentration in this stratum is kept low (Figure 8A).

During aging, a still not completely understood breakdown of the epidermal calcium gradient takes place [3]. The calcium in the epidermis is not lost, but its concentration increases in the *stratum basale* and decreases in the *stratum granulosum*. This has two consequences: (1) a less pronounced epidermal barrier with e.g. reduced levels of loricrin; (2) a reduced proliferation rate of progenitor cells in the *stratum basale* (Figure 8B). In the course of this work, we could demonstrate that a rise of the calcium concentrations doubles the time of the cell cycle (from 27 hours to 66 hours) in HaCaT cells.

To cope with this altered calcium distribution the epidermis reacts with an increased expression of miRNAs as hsa-mir542-5p, hsa-mir125a-5p, hsa-mir135a-5p, hsa-mir196a-5p, hsa-mir491-5p and hsa-mir552-5p. We could show here that in the skin of elderly people the amount of these miRNAs increases at least 35-fold. The highest expression level was found for mir542-5p with a 200-fold upregulation in aged skin. The localization of the latter miRNA was studied in an RNA ISH approach. In fact, the presence of mir542-5p in the *stratum basale* was corroborated as well as the high expression level in the skin of elderly people. In cell culture experiments after transduction of HaCaT cells we could demonstrate that mir542-5p counterbalances the calcium induced slow-down of the cell cycle (Figure 8B). These data fit quite well to results published by the Grillari group previously [27]. They showed that senescent skin fibroblasts as part of the senescence-associated secretory phenotype significantly secrete hsa-mir542-5p, hsa-mir196a-5p, hsa-mir125a-5p and mir491-5p and pack them into small extracellular vesicles [27]. It is therefore quite possible that the fibroblasts deliver these vesicle packed miRNAs to the epidermis in aged skin and thus modulate proliferation and differentiation of keratinocytes.

The increased levels of the miRNAs mentioned above have a major drawback for the outermost layers of the epidermis: For example, the small non-coding RNA mir542-5p is not specifically localized to the *stratum basale* but can be found in all other epidermal layers. Especially in the *stratum granulosum* and *corneum,* these RNA molecules can be harmful for the skin and could result in a breach of the epidermal barrier (Figure 8C). A transfection of HaCaT cells with mir196a-5p and mir542-5p revealed decreased mRNA levels of most CE precursor family members. By an unknown mechanism, the RNA degradation mediated by mir542-5p is strictly dependent on the presence of high amounts of calcium.

To prevent damage to the skin a compensation mechanism based on a lncRNA was established (Figure 8D). We could show that during skin aging SPRR2C levels, a lncRNA that arose during hominid evolution, are increased. Bioinformatics analysis revealed that SPRR2C is targeted by mir542-5p, mir125a-5p, mir135a-5p, mir196a-5p, mir491-5p and mir552-5p. Of all these miRNAs, mir542-5p is the best candidate and thus showed the lowest mfe upon binding to SPRR2C. It also has to be mentioned that these miRNAs are not specific for SPRR2C but target the mRNAs of most CE precursor family members. In this way, SPRR2C acts as a decoy for miRNAs and thus promotes the establishment of the CE and the epidermal barrier as a whole. Previously, binding of mir196a-5p to SPRR2C was demonstrated [23]. By applying a reporter approach based on firefly and renilla luciferases, we could confirm a binding of mir196a-5p and mir542-5p to SPRR2C. A qPCR analysis also demonstrated that after transfection of HaCaT cells with either mir196a-5p or mir542-5p levels of SPRR2C are drastically reduced.

Opposing to miRNAs SPRR2C is promoting keratinocyte differentiation. Overexpression of SPRR2C in cell culture experiments in fact resulted in increased RNA levels of most CE precursor family members. SPRR2C itself is not promoting cell differentiation in the *stratum basale*, because this lncRNA is strictly localized to the *stratum granulosum/corneum* where the transition from keratinocytes to corneocytes takes place. This result also stresses the importance of gradients and localizations of proteins, ions and RNAs in the epidermis. To promote keratinocyte differentiation calcium as well as lncRNAs are concentrated in the outermost layers of the skin. As an exception to this rule, miRNAs are evenly distributed over all epidermal layers. In the *stratum basale* this small non-coding RNAs facilitate cell proliferation. In the *stratum granulosum* their action is nullified by the lncRNA SPRR2C. Furthermore, it would be interesting to see if these miRNAs fulfil a specific action in the *stratum spinosum* during skin aging.

## MATERIALS AND METHODS

### miRNA prediction

miRNAs that target SPRR2C were predicted using microrna.org based on the mirSVR regression method [28, 29], Microcosm Targets based on the TargetScanS algorithm [30], miTarget [31] and MicroTar [32].

### Human skin samples

Human female eyelids were obtained after routine plastic surgery from the hospital “Barmherzige Brüder” (Salzburg, Austria), foreskin samples from the Department of Urology and Children ′s Surgery of the SALK/PMU Salzburg. All skin samples were non-inflamed, and patients gave their written consent. The study was conducted in accordance with the Declaration of Helsinki, and the protocol was approved by the Ethics Committee of the government of Salzburg, Austria (415-EP/73/548-2015).

### RNA isolation

In case of cell culture experiments, the cells were trypsinized, centrifuged and washed with pre-cooled 1xPBS, pH 7.4. The cell pellet was resuspended in 1 ml peqGOLD TriFast ™ (VWR) for RNA-Isolation. In case of human skin tissues, the skin samples were homogenized in peqGOLD Trifast using the Ultra-Turrax T8 (IKA). Afterwards, total RNA was isolated according to the manufacturer′s protocol.

### RT-PCR for mRNA

1 μg RNA was mixed with anchored oligo-dT-primers (0.5 mmol/μl) and a transcription of RNA into cDNA was performed using either Lucigen (Biozym) or MMLV High Performance Reverse Transcriptase (Epicentre) according to the manufacturer’s instructions. Primers were designed using the online available primer 3 software (http://primer3.ut.ee/) and were ordered from Sigma-Aldrich. The primer sequences can be found in Supplementary Table 1. The analysis was performed using either a GoTaq^®^ qPCR Master Mix from Promega (Madison) or a Qiagen SYBR-Green mix (QIAGEN) on a Rotor-Gene Q (Qiagen, Hilden). The following settings were used: 1 cycle: 95° C 240 s; 40 cycles: 95° C 10 s, 65° C 15 s, 72° C 15 s, and 72° C for 300 s. To create melting curves, the PCR mixes were heated from 65° C to 95° C. The acidic ribosomal protein ARP (RPL0) was used for normalization. The fold-changes were calculated using the formula 2^−(ΔΔCt)^.

### RT-PCR for miRNA

The miRNAs were quantified as described in [33]. 5 μg RNA was polyadenylated by an *E.coli* Poly(A) Polymerase (NEB; M0276) according to the manufacturer′s instructions. After phenol/chloroform extraction and ethanol precipitation, a reverse transcription with 1μg of the modified RNA was performed using MMLV High Performance Reverse Transcriptase (Epicentre) according to the manufacturer’s protocol. The RT primer had the following sequence: 5′-GCG AGC ACA GAA TTA ATA CGA CTC ACT ATA GG(T)12N (N=A, G, C). For RT-PCR with the same conditions as above the universal primer with the sequence 5′-GCG AGC ACA GAA TTA ATA CGAC-3′ as well as one of the following sense primers were used: hsa-mir542 (5′-TCG GGG ATC ATC ATG TCA CGA GA-3′); hsa-mir196a (5′-TAG GTA GTT TCA TGT TGT TGG G-3′); hsa-mir491 (5′-AGT GGG GAA CCCT TCC ATG AGG-3′); hsa-mir125a (5′-ACA GGT GAG GTT CTT GGG AGC C-3′); hsa-mir135a (5′-TAT AGG GAT TGG AGC CGT GGC G-3′); hsa-mir552 (5′-GTT TAA CCT TTT GCC TGT TGG-3′). The acidic ribosomal protein ARP (RPL0) was used for normalization.

### Cell culture and transfections

HaCaT cells [34] were grown in T25 cell culture flasks containing DMEM supplemented with 10% fetal calf serum (FCS; Life Technologies/Gibco) and 1% penicillin/streptavidin at 37° C, 5% CO_2_ and humidified atmosphere. HEK293FT cells were cultivated under the exact same conditions, but non-essential amino acids were added. Medium was changed every 2-3 days. For subculturing cells were split once a week at a confluence of approximately 80 %. Lipofection was performed at a confluency of approximately 90% in 12-well plates. 300 ng vector (either psicheck2, SPRR2C-vector or SPRR2C407-628-vector), 10-20 nM miRNA (either MISSION miRNA MIMIC hsa-mir196a (HMI0322; Sigma-Aldrich), MISSION miRNA MIMIC hsa-mir542 (HMI0725; Sigma-Aldrich) or MISSION miRNA MIMIC Negative Control #1 (HMC0002; Sigma-Aldrich)) were transfected into HepG2 cells using Roti®fect (Carl Roth) according to the manufacturer′s instructions. Cells were incubated for 24 hours at 37° C, 5% CO_2_ and humidified atmosphere prior to further processing. miRNA transfection efficiency was determined using qPCR.

### *In situ* hybridization

Cryosections of the skin samples (thickness 10 μm) were dried at RT (10-20 min), fixed in 4% paraformaldehyde in phosphate buffered saline/tween (PBT) (PBS supplemented with 0.1% (v/v) Tween 20) (15 min), and rinsed in PBT (3x5 min). Acetylation occurred in 0.1 M triethanolamine (pH 8.0) with 2.5 μl/ml acetic anhydride (10 min), and was followed by a wash in PBT (5 min) and dehydration in a graded ethanol series (50%, 70%, 3x 100%, 3 min each). Hybridization buffer composed of 1x salt solution (150 mM NaCl; 5 mM PIPES; 5 mM EDTA; pH 6.8) and 1x Denhardt’s solution (0.02% BSA; 0.02% Ficoll; 0.02% polyvinylpyrrolidone), 50% deionised formamide, 10% dextran sulphate and 1 mg/ml yeast RNA, was used for prehybridisation treatment (2 h) and then replaced by hybridisation buffer containing 0.25 μl/ml digoxigenin-labelled RNA probe (SPRR2C (5′-TCT TTG ATG GCA CCC AAA GAG AAG CTC TGA AAG GCC GGT ACA GCT G-3′) and mir542-5p (5′-TCT CGT GAC ATG ATG ATC CCC GA-3′) for hybridisation overnight. For prehybridisation and hybridisation, the slides were covered with strips of parafilm and placed horizontally in a wet chamber at 50° C. Post-hybridisation washes were carried out in prewarmed (65° C) wash solution (50% deionised formamide; 2x saline sodium citrate (750 mM NaCl; 75 mM tri-sodium citrate; pH 7.5); 0.1% Tween 20, 1x a few s, 2x45 min), and in MABT (100 mM maleic acid; 150 mM NaCl; 0.1% Tween 20; pH 7.5; 2x45 min, at RT). Sections were then encircled with a wax pen (DAKO), blocked with 20% normal goat serum and 2% Boehringer Mannheim blocking reagent in MABT (90 min), and reacted with anti-DIG alkaline phosphatase antibody (1:2000 in blocking solution, in a wet chamber overnight, at RT). After washing in MABT (8x20 min) and in alkaline phosphatase buffer (100 mM Tris-HCl, pH 9.5; 100 mM NaCl; 25 mM MgCl2; 2 mM levamisole; 0.1% Tween 20; 3x10 min, at RT) sections were stained with NBT (5 μl/ml in alkaline phosphatase buffer also containing 3.75 μl/ml X-phosphate, at RT in the dark). Colour development was stopped by washing 3x in PBT containing 1 mM EDTA and 2x in double-distilled water. Sections were mounted in Gel/Mount aqueous embedding medium. Photographs of the stained specimens were taken on a Reichert Polyvar microscope.

### Cloning

The chromosomal SPRR2C region was PCR amplified from human genomic DNA using the following primers: fwd (CAC CGC TAG CAG AGG TTC TCA GCC TCA TGA CTC CTA AAC); rev (CTA GTG TAC ACC TGT ATT AGT TAT ATC CAA). The resulting PCR product was cloned into pLL3.7 using BsrGI and NheI. Full length SPRR2C mRNA was PCR amplified from human cDNA using the primers fwd 5′-CCG CTC GAG AGG TTC TCA GCC TCAT GAC TCC TA-3′ and rev 5′-ATA AGA ATG CGG CCG CCA TAG CTA CTT TAT TCA GGG-3′ and cloned into the vector psiCHECK2 using XhoI and NotI. A region of SPRR2 mRNA (407-628) was PCR amplified from the vector above using fwd (5-′CCG CTC GAC AGG GAG CTT CTT TCC CCT T-3′) and rev (5′-AAG GAA AAA AGC GGC CGC GAT AGT AGA AGC TCA TGC CA-3′) and cloned into the psiCHECK vector using XhoI and NotI. A region of SPRR2C (281-486) was PCR amplified using fwd (5′-TTA ATA CGA CTC ACT ATA GGA AAG TGT CCA CCC AAG AG-3′) and rev (5′-CAC CTT GGA AGC CAT GGT GGG GCA GCC TCA GAA AGG AAA C-3′) and cloned into the NheI linearized vector psiCHECK2™ using the Gibson Assembly® Master Mix (NEB, E2611).

### Transfection and virus production

A detailed protocol is given in [25]. Briefly summarized, HEK 293FT cells in a 75 cm^2^ flask at a confluency of 95% were changed to fresh DMEM medium supplemented with 10% fetal bovine serum 2 hours before transfection. A solution containing 5 μg pMD2G, 10 μg pMDLg/pRRE, 5 μg pRSV-Rev, 20 μg pLL3.7 [35] (with or w/o SPRR2C) and 250 mM CaCl2 in a volume of 800 μl were added dropwise to 800 μl 2× HBS (pH 7.0) under constant vortexing. After a 30 min incubation at room temperature the solution was added dropwise to the 293FT cell monolayer. At the following two days the supernatant was collected containing the virus particles. After filtration trough a 45 μm sterile filter the supernatant was directly added to HaCaT cells grown to 60% confluency and pretreated with 8 μg/mL polybrene. After 48h the viral supernatant was exchanged for DMEM medium supplemented with 10% FBS and cultured until further use.

### Western blot analysis

Transduced HaCaT cells were lysed by addition of a CE extraction buffer (0.0625 M Tris-Cl, 5%SDS, 10% glycerol, 20% beta-mercaptoethanol, pH 6.8). The samples were heated to 95° C for 15 min. Equal amounts were loaded onto a 15% SDS-PAGE gel and were blotted on a nitrocellulose membrane using the following parameters: 250 mA, 90 min at RT. MTBS-T (25mM TRIS pH 7.6, 137 mM NaCl, 0.1% TWEEN 20, 5% nonfat milk powder) was used for blockage for 90 min at RT. The membranes were washed with TBS-T for 30 min and then the primary-antibodies were added and incubated over night at 8° C. The following dilutions were used: anti-SPRR2A (rabbit) (Acris; AP54034PU-N) 1:500 dilution; anti-Annexin A1 1:500).

After three TBS-T wash steps (10 min at RT) either an alkaline phosphatase conjugated affinity pure goat anti rabbit IgG Fc Fragment specific antibody (111-055-003; Jackson, West Grove, PA) in a 1:5000 dilution was added. After a 4 hour incubation at RT and three TBS-T wash steps the detection was performed using the Western Blue® stabilized substrate (Promega, S3841).

### Dual-luciferase assay

Immortalized keratinocytes were grown in KGM-Gold growth medium (Lonza, 00192060) in 12-well plates supplemented with 0.09 mM CaCl_2_ till a confluence of 70-80% was reached. The growth medium was removed and the transformation mix (125 nM miRNA (either MISSION miRNA MIMIC Negative Control #1 or MISSION miRNA MIMIC hsa-mir542); 350 ng psiCHECK2™-SPRR2C (281-486) and 2.3 μl TransFast™ transfection reagent (Promega, E2431). After an incubation for 60 min at 37° C KGM-Gold growth medium supplemented with 0.8 mM CaCl_2_ was added. The growth medium was removed after 48 hours and the cells were lysed with the passive lysis buffers provide by the Dual-Luciferase® Reporter Assay System (Promega, 1910). The further assay was performed according the manufacturer′s instructions by using the GloMax® Explorer Multimode Microplate Reader (Promega, GM3500). After addition of 100 μl Luciferase Assay Buffer II the firefly luciferase activity was measured, after addition of 100 μl Stop and Glo® Buffer the renilla luciferase activity was measured.

### Statistical analysis

The Shapiro-Wilk Test was used as a test of normality. Data in Figures 1A, 5B, 6 were analysed by a unpaired one-way analysis of variance (ANOVA) followed by a TUKEY post hoc test. Data in Figures 3, 4, 5A were analyzed by an independent t-test analysis. *: p<0.1; **: p< 0.05; ***: p<0.01. Microarrays were analyzed by the Mann-Whitney U tests for independent samples. Most data are displayed as arithmetic means ± standard deviation.

## Supplementary Material

Supplementary Figures

Supplementary Table 1
