# Supplementary material for: The life in a gradient: calcium, the lncRNA SPRR2C and mir542/mir196a meet in the epidermis to regulate the aging process
Source: Aging (Albany NY). 2021 Aug 2;13(15):19127–44. doi: 10.18632/aging.203385 (PMC8386546; doi:10.18632/aging.203385)
Supplement: Supplementary Figures [file aging-13-203385-s001.pdf]

SUPPLEMENTARY FIGURES

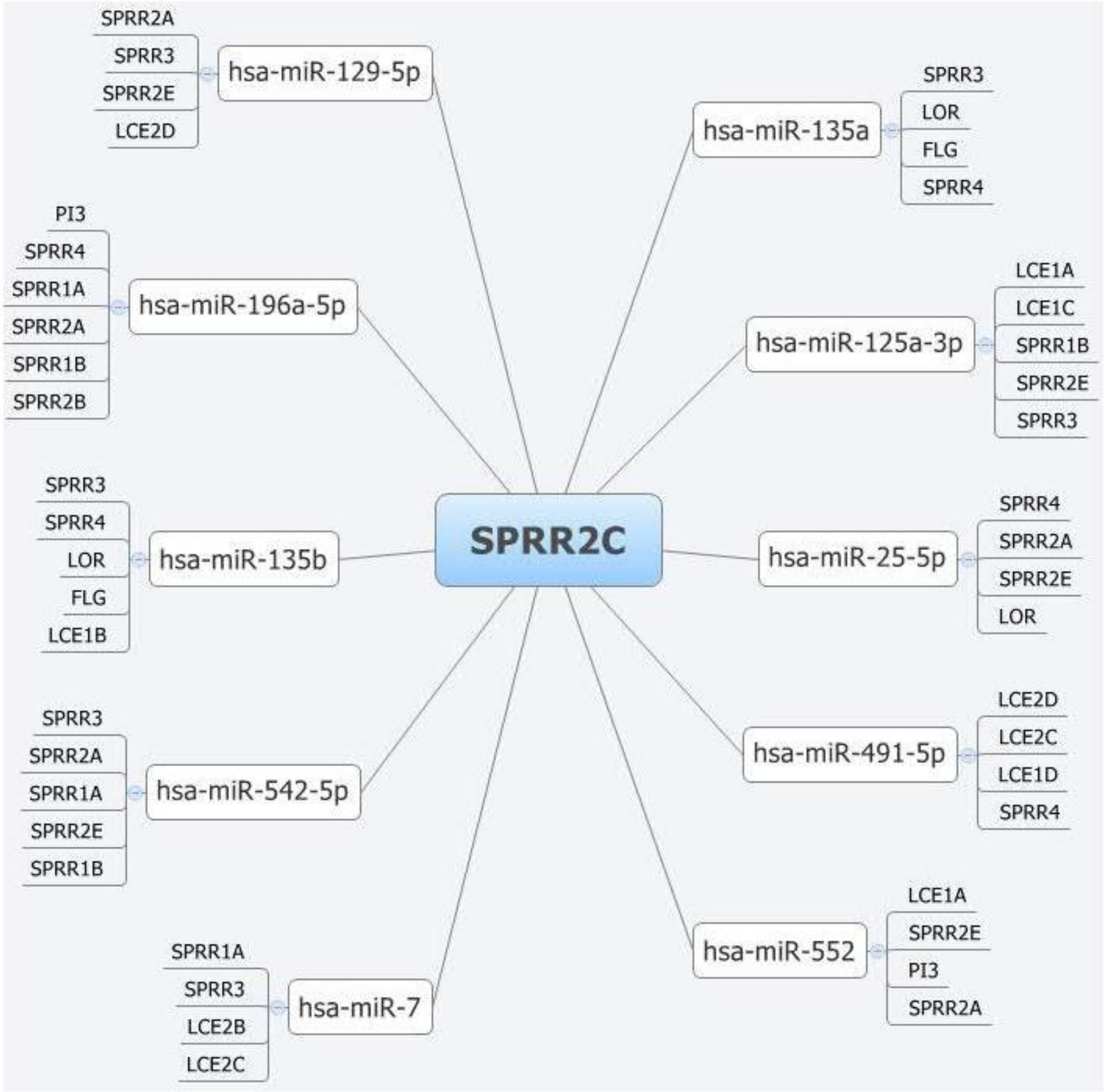

Supplementary Figure 1. The miRNAs hsa-mir129-5p, hsa-mir196a-5p, hsa-mir135b, hsa-mir542-5p, hsa-mir7, hsa-mir135a, hsa-mir125a-3p, has-mir25-5p, hsa-mirmir491-5p and has-mir552 bind do SPRR2C as well as to other members of the CE precursor family.

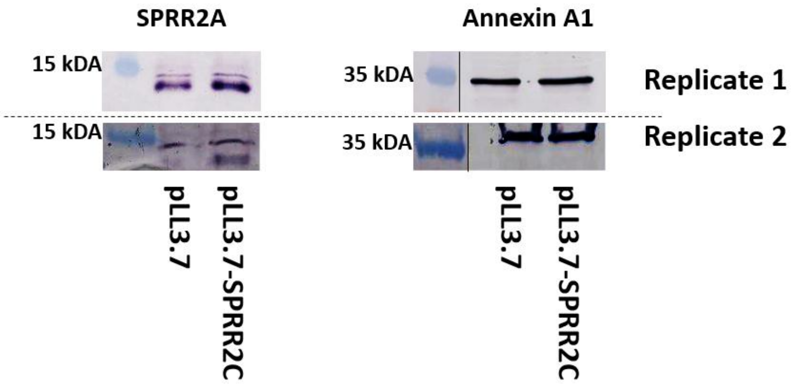

Supplementary Figure 2. A Western blot analysis with an SPRR2A antibody confirms an increased expression of this protein (7.965 kDA) in HaCaT cells after transduction with pLL3.7-SPRR2C compared to HaCaT cells transduced with the empty control vector pLL3.7. In Replicate 1 a 1.42-fold increase was observed and in replicate 2 a 33-fold increase was observed. (A third replicate revealed a 1.59-fold increase in SPRR2A levels (data not shown)). A Western blot with an Annexin A1 antibody (MW: 37 kDa) demonstrates that equal amounts were loaded onto the 15% polyacrylamide gel.
