# Supplementary material for: The life in a gradient: calcium, the lncRNA SPRR2C and mir542/mir196a meet in the epidermis to regulate the aging process
Source: Aging (Albany NY). 2021 Aug 2;13(15):19127–44. doi: 10.18632/aging.203385 (PMC8386546; doi:10.18632/aging.203385)
Supplement: Supplementary Table 1 [file aging-13-203385-s002.pdf]

## SUPPLEMENTARY TABLE

**Supplementary Table 1. Primer sequences for RT-PCR.**

| | | |
|---|---|---|
| ARP | fwd | 5´-GGC ACC ATT GAA ATC CTG AGT GAT GTG-3´ |
| | rev | 5´-TTG CGG ACA CCC TCC AGG AAG C -3´ |
| EVPL | fwd | 5´-AAG GAA CGG CTG AGC TAC AA-3´ |
| | rev | 5´-AAA TAA CCA CCC GCA GTG TC-3´ |
| IVL | fwd | 5´-AAA TAA CCA CCC GCA GTG TC-3´ |
| | rev | 5´-ATC GCT CCT TTG CAC CTC TA-3´ |
| LCE1A | fwd | 5´-CTG CAA GAG TGG CTG AGA TG-3´ |
| | rev | 5´-GCA GGA AAT GAG CTG GAG AG-3´ |
| LCE2A | fwd | 5´-CCC GAT TGT TGT GAG TGT GA-3´ |
| | rev | 5´-AGA AAA AGG GGC AGG AGA AG-3´ |
| LCE2B | fwd | 5´-CCG ACT GCT GTG AGA GTG AA-3´ |
| | rev | 5´-AAG CCC CAT GAG TCT TTG TG-3´ |
| LCE3C | fwd | 5´-AGT TGT CCC TCA CCC AAG TG-3´ |
| | rev | 5´-ATT GAT GGG ACC TGA AGT GC-3´ |
| LCE6A | fwd | 5´-TGA GGC ACA GGT GCA GTT TAC -3´ |
| | rev | 5´-CTG AA GGG GTG AAT GGA AGA -3´ |
| LOR | fwd | 5´-GGA GTT GGA GGT GTT TTC CA-3 |
| | rev | 5´-ACT GGG GTT GGG AGG TAG TT-3´ |
| PPL | fwd | 5´-TGG ATT GTG CTG CTG TTA GC-3´ |
| | rev | 5´-GAC TCC AGC CAC CAG GTT TA-3´ |
| SPRR1B | fwd | 5´-CAT TCT GTC TCC CCC AAA AA-3´ |
| | rev | 5´-ATG GGG GTA TAA GGG AGC TG-3´ |
| SPRR2A | fwd | 5´-CCT GAC AGC AAA AAG TTT CT-3´ |
| | rev | 5´-AAA GGT GGT AGA AGC TCG-3´ |
| SPRR2B | fwd | 5´-GCC AAA GTA TCC ACC GAA GA-3´ |
| | rev | 5´-AAC ATC ATG GGC AGA TCA CA-3´ |
| SPRR2G | fwd | 5´-TCC ACC ATG CCA GGA TAA AT-3´ |
| | rev | 5´-GCT GAA GGG AAG ATG ATG GA-3´ |
| SPRR2E | fwd | 5´-ATT GGC TCA CCT TGT TCC AC-3´ |
| | rev | 5´-TGG GAA CTG ACA CTG CTG AG-3´ |
| SPRR2F | fwd | 5´-TGA TAG CAA AAG GTT TTC TTT T-3´ |
| | rev | 5´-TTA TTC AGG GAG TGA AAG GA-3´ |
| SPRR3 | fwd | 5´-TCA GCA GAA GAC CAA GCA GA-3´ |
| | rev | 5´-GAG ACT ATG GCT TGG GGT GA-3´ |
| SPRR4 | fwd | 5´-CTC CTC CTG CCC AAG ATG TA-3´ |
| | rev | 5´-TTC CTC ACT TTG GGA AAT GG-3´ |
| SPRR2C | fwd | 5-AGC CAT CCA GGG ATA CAC AG-3´ |
| | rev | 5´-CCG GTA CAG CTG AGG ACT TC-3´ |
| Renilla | fwd | 5´-TCG TCC ATG CTG AGA GTG TC-3´ |
| | rev | 5´-CTC CAG TTT CCG CAT GAT CT-3´ |
| Firefly | fwd | 5´-TTC GCT AAG AGC ACC CTG AT-3´ |
| | rev | 5´-GTA ATC AGA ATG GCG CTG GT-3´ |
